# Virologic versus immunologic monitoring and the rate of accumulated genotypic resistance to first-line antiretroviral drugs in Uganda

**DOI:** 10.1186/1471-2334-12-381

**Published:** 2012-12-27

**Authors:** Steven J Reynolds, Hakim Sendagire, Kevin Newell, Barbara Castelnuovo, Immaculate Nankya, Moses Kamya, Thomas C Quinn, Yukari C Manabe, Andrew Kambugu

**Affiliations:** 1 Division of Intramural Research, National Institute of Allergy and Infectious Diseases, National Institutes of Health, Bethesda, MD, USA; 2 Johns Hopkins University School of Medicine, Baltimore, MD, USA; 3 Uganda Ministry of Health, Kampala, Uganda; 4 Department of Microbiology, Makerere University College of Health Sciences, Kampala, Uganda; 5 Clinical Research Directorate/Clinical Monitoring Research Program, SAIC-Frederick, Inc, Frederick National Laboratory for Cancer Research, Frederick, MD, 21702, USA; 6 Infectious Diseases Institute, Makerere University College of Health Sciences, Kampala, Uganda; 7 Joint Clinical Research Center, Kampala, Uganda; 8 NIAID/NIH ICER Program, P.O. Box 7007, Kampala, Uganda

**Keywords:** HIV-1, Antiretroviral therapy, Drug resistance

## Abstract

**Background:**

Viral load monitoring (VLM) to identify individuals failing antiretroviral therapy (ART) is not widely available in resource-limited settings. We compared the genotypic resistance patterns between clients with VLM versus immunological monitoring (IM).

**Methods:**

Between 2004–2008, 559 ART naïve clients were enrolled in a prospective cohort, initiated on ART, and monitored with viral load (VL) and CD4+ cell counts every 6 months (VLM group). From February 2008 through June 2009, 998 clients on ART for 36–40 months (corresponding to the follow-up time of the VLM group) at the same clinic and monitored with CD4+ cell counts every 6 months were recruited into a cross sectional study (IM group). Samples from VLM clients at 12, 24 and 36 months and IM clients at 36–40 months with VL > 2000 copies/ml underwent genotypic drug resistance testing.

**Results:**

Baseline characteristics were similar. Virologic failure (VL > 400 copies/ml) at 12, 24 and 36 months in the VLM group were 12%, 6% and 8% respectively, and in the IM group 10% at 36–40 months. Samples from 39 VLM and 70 IM clients were genotyped. 23/39 (59%) clients in the VLM group (at 12, 24 or 36 months) compared to 63/70 (90%) in the IM group, (*P* < 0.0001) had at least 1 non-nucleoside reverse transcriptase mutation. 19/39 (49%) of VLM clients had an M184V mutation compared to 61/70 (87%) in the IM group (*P* < 0.0001). Only 2/39 (5%) of VLM clients developed thymidine analogue mutations compared to 34/70 (49%) of IM clients *(P <* 0.0001).

**Conclusions:**

Routine VL monitoring reduced the rate of accumulated genotypic resistance to commonly used ART in Uganda.

## Background

Antiretroviral treatment (ART) programs have scaled up to reach over 5.2 million HIV-infected individuals in need of life-saving treatment in low and middle income countries by the end of 2009 [1]. Most of these individuals live in settings where laboratory monitoring is limited and treatment failure is determined using either clinical or immunological criteria. Concern has been raised regarding the performance of clinical and immunologic treatment failure criteria to correctly identify individuals with virologic failure [2-7]. Prolonged virologic failure may result in accumulation of resistance mutations to commonly used first-line ART as observed in Malawi and elsewhere [8,9]. This could compromise second-line treatment outcomes and also increase the risk of transmitted HIV drug resistance.

Failure to achieve or maintain virologic suppression for prolonged periods of time may lead to the sequential development of thymidine analogue mutations (TAMs) in clients taking nucleoside reverse transcriptase inhibitors (NRTIs) such as zidovudine (ZDV) or stavudine (d4T) as part of their ART regimens [9-12]. The accumulation of 3 or 4 TAMs confers drug resistance across the NRTI class of antiretroviral (ARV) drugs and has implications for future ART regimens, particularly in resource-limited settings (RLS) where second-line ART options are limited. ZDV, tenofovir (TDF) and to a lesser extent d4T are currently used as the nucleoside backbone of the first-line and some second-line regimens in sub-Saharan Africa and other RLS areas.

The objective of our study was to compare genotypic resistance patterns (with particular attention to TAMs) among a group of clients on first-line ART at an urban clinic in Kampala, Uganda, who were monitored both clinically, immunologically and virologically as part of a research cohort study to a similar group of clients attending the same clinic who were monitored only clinically and immunologically.

## Methods

### Study setting

The Infectious Diseases Institute (IDI) of Makerere University College of Health Sciences (Kampala, Uganda) is a center of excellence in the delivery of HIV clinical care, research and training, with over 10,000 active clients registered including over 6,500 receiving ART. Eligibility criteria for ART at the time of this study (February 2008 through June 2009) were CD4 < =200 cells/ul or WHO stage IV disease; first-line regimens included stavudine or zidovudine plus lamivudine and nevirapine or efavirenz.

### Study population

#### The IDI routine care clinic (immunological monitoring (IM) group)

Clients attending the routine care clinic who become eligible for ART are offered adherence counseling prior to ART and also during follow-up if deemed necessary by clinicians. Clients are seen monthly for follow-up assessments, drug refills and monitored through prospective CD4+ cell counts every 6 months. Treatment failure was assessed according to the WHO immunologic or clinical criteria for treatment failure [13]. A more detailed description of this population is presented elsewhere [14].

#### The IDI research cohort (viral load monitoring (VLM) group)

A nested research cohort within the routine care clinic was established in 2004, with a consecutive prospective enrollment of 559 ART-naïve clients who initiated ART from April 2004 through April 2005; clients enrolled in this cohort are monitored through both CD4+ counts and viral load measurement (Amplicor HIV-1 Monitor PCR Test, version 1.5; Roche Diagnostic, GmbH Molecular Systems, Pleasanton, CA with a detection limit of 400 copies/ml), every 6 months; plasma is also stored for future investigations. A more detailed description of the study procedures and data collection has been presented elsewhere [15]. In brief, ART-eligible adults (> = 18 years of age) were enrolled in the study if they fulfilled the following eligibility criteria: 1) confirmed HIV type 1 infection; 2) regular attendance at clinic visit, based on at least 2 clinic visits within the previous 6 months; 3) stable residence within a 20-km radius of Kampala; 4) willingness to be followed at the Infectious Diseases Institute for at least 2 years; and 5) eligibility for ART according to the World Health Organization (WHO) 2003 and Uganda Ministry of Health (ie, a CD4+ cell count < = 200 cells/ul or WHO stage IV), and 6) provision of written informed consent. Clients were seen every three months by the study clinicians and monthly for routine clinical visits and drug refills. Clients were considered for switch to second line ART if they had two HIV VL > 1000 copies/ml on two consecutive measurements.

### Study design

From February 2008 through June 2009, a cross sectional study was conducted in the IDI routine care clinic population recruiting a convenience sample of clients who were on first-line ART regimens for 36–40 months (corresponding to the length of follow-up time reached by IDI research cohort clients). Clients still on first-line ART were consecutively recruited during their monthly follow-up visits. After providing written informed consent, blood was drawn for HIV viral load (VL), CD4+ cell count and plasma was stored for genotyping. Genotyping was done on all clients found to have an HIV VL > 2,000 copies/ml (corresponding to the sensitivity threshold of the assay). Results of the viral load and genotyping test were made available to the physicians for clinic management of their clients.

In the research cohort clients, those alive and in care at 12 months were considered eligible for this analysis. We included any research cohort client having a HIV VL of >2000 copies/ml while still on first-line at months 12, 24 or 36 of follow-up; however, clients who had a detectable VL above 2,000 copies/ml up to month 12 but subsequently (months 24 or beyond) became virologically suppressed without regimen change were not included in the genotyping analysis as these were considered to have early adherence problems rather than drug resistance [16]. All clients in both groups were followed-up regardless of treatment change. Genotyping was done retrospectively on stored plasma samples. Genotypic mutation patterns of the first occurrence of a viral load >2,000 copies/ml in the research cohort clients and the mutation patterns observed among in the routine clinic population at 36–40 months were compared. All genotypes reported on VLM clients were obtained prior to switch to second-line treatment.

### Statistical methods

Baseline characteristics for both monitoring groups were compared using the Fisher exact test for categorical variables (gender, WHO staging, ART regimen) and Mann–Whitney U test for continuous variables (age, CD4+ count, hemoglobin, body mass index). We used the Mann–Whitney U test to compare median CD4+ count increase at follow-up. We used the t-test for proportions to assess differences in mutation rates across monitoring groups. All *P* values are two-sided. All analyses were performed using SAS version 9.2 (SAS Institute, Cary, NC) or Stata version 11.1 (StataCorp, College Station, TX).

### Laboratory procedures

Plasma was separated within 2 hours from blood draw and stored immediately at −80°C. HIV-1 RNA was tested with the Roche Amplicor MONITOR v1.5 assay (Roche Molecular Systems, Nutley, New Jersey, USA). HIV-1 RNA was extracted from plasma samples using a Qiagen RNA extraction method (Qiagen Inc., Chatsworth, California, USA). Polymerase gene-specific primers were used for reverse transcriptase, followed by 750 base pair pol gene encompassing amino acids 1–242 of reverse transcriptase. The PCR products was then purified using the QIAquick PCR purification kit (Qiagen, Valencia, California, USA) and sequenced using Beckman Coulter sequencing kit (Beckman Coulter Inc., Fullerton, CA). The obtained sequences were then edited using BioEdit sequence editor version 7.0.4. The cleaned sequences were then uploaded into the Stanford University HIV Drug resistance database to obtain the drug resistance profile. Genotyping assays were performed at the Joint Clinical Research Center laboratory in Kampala. For genotype analysis, mutations were generally categorized according to the International AIDS Society-USA recommendations [17].

### Ethics

All study participants from the IDI routine care clinic provided written informed consent, while the participants from the IDI research cohort had already provided consent at study enrollment for future use of stored plasma. The study was approved by the National AIDS Research Council Ethics Board, the Uganda National Council for Science and Technology, and the National Institute of Allergy and Infectious Diseases Intramural Institutional Review Board.

Genbank accession numbers: KC008609 to KC008711.

## Results

Four hundred forty one of the 559 research cohort clients (VLM group) were alive and in care at 12 months and included in this analysis. Nine hundred ninety-eight clients on ART for 36–40 months were recruited from the routine clinic population (IM group). Table 1 displays the demographic characteristics for the VLM and IM clients. Characteristics of patients in the VLM and IM group at ART initiation were similar; particularly median baseline CD4+ cell count was 102 (IQR 31–170) cells/μl for VLM clients and 86 (29–154) for IM clients. The median CD4+ change from baseline to 36 months was 227 (146–336) for VLM clients and 251 (148–377) for IM clients; virologic failure rates (VL > 400 copies/ml) at 12, 24 and 36 months were 12%, 6% and 8% respectively for VLM clients and 10% at 36–40 months for IM clients.

**Table 1:** Baseline Characteristics

**Characteristic**	**IM (n = 998)**	**VLM (n = 441)**	**P-value**
Age (IQR yrs)	36.2 (31.5 – 41.3)	35 (30 – 41)	0.002
Gender			0.667
Female	680 (68%)	306 (69%)	
Male	318 (32%)	135 (31%)	
Baseline CD4 (IQR cells/μl)	86 (29–154)	101.5 (30.5-170)	0.330
WHO Stage	(n = 996)		<0.0001
WHO Stage I	18 (2%)	2 (1%)	
WHO Stage II	233 (23%)	49 (11%)	
WHO Stage III	478 (48%)	257 (58%)	
WHO Stage IV	267 (27%)	133 (30%)	
Hb (IQR)	11.3 (10.0 – 12.7)	11.7 (10.6 – 13.0)	0.001
BMI (IQR kg/m^2^)	20.2 (18.2 – 22.5)	20.2 (18.3 – 22.6)	0.917
ART Regimen			0.726
d4T/3TC/NVP	721 (72%)	325 (74%)	
d4T/3TC/EFV	3 (0.5%)	0	
ZDV/3TC/NVP	2 (0.5%)	0	
ZDV/3TC/EFV	272 (27%)	116 (26%)	

IM: Immunologically monitored; VLM: Virologically monitored; IQR: Interquartile Range; Hb: Hemoglobin; BMI: Body mass index; ART: antiretroviral therapy; d4T: stavudine; 3TC: lamivudine; ZDV: zidovudine; NVP: nevirapine; EFV: efavirenz.

**Figure 1:**
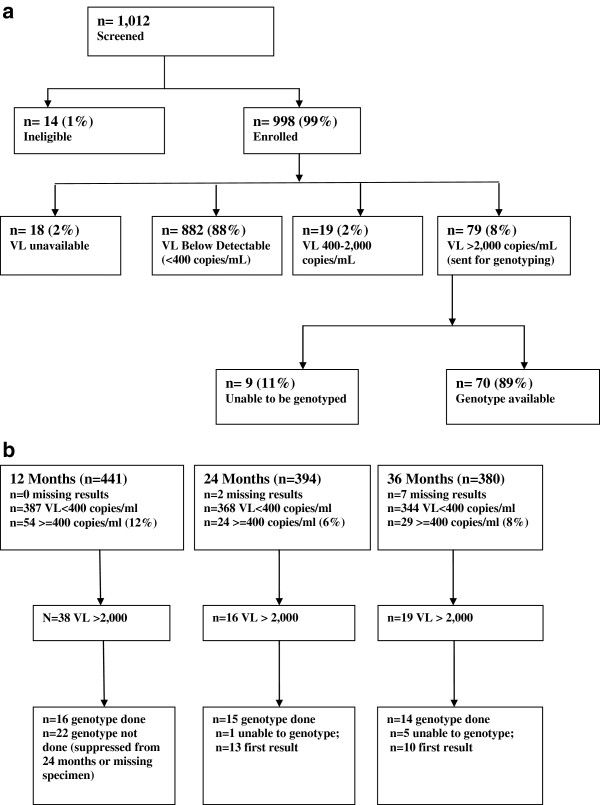
a. Enrollment and accrual: Immunologically monitored clients. b. Virologically monitored clients. VL: Viral Load.

Samples from 39 VLM and 70 IM clients were successfully amplified and genotyped (Figure 1a &1b). Some VLM clients had more than one time point sent for genotyping resulting in a total of 45 unique genotypes for this group; among clients with >1 genotype, the first available result was used to compare VLM and IM clients as this was considered to be the time point when a switch of therapy would be considered. Among VLM clients, rates of resistance were relatively stable over 36 months follow-up (Table 2). The most common class of resistance was to the non-nucleoside reverse transcriptase inhibitors (NNRTIs) ranging from 50% (8/16) at 12 months, 69% (9/13) at 24 months and 60% (6/10) at 36 months. Resistance to lamivudine was present among 50% (8/16) at 12 months, 62% (8/13) at 24 months and 40% (4/10) at 36 months. Very few VLM clients developed any resistance to the nucleoside reverse transcriptase inhibitors (NRTIs) apart from the M184V mutation.

**Table 2:** Resistance over time for virologically monitored (VLM) clients

	**Month 12**	**Month 24**	**Month 36**
	**(N = 16)**	**(N = 13)**	**(N = 10)**
**NRTI mutations**			
Any TAMs	2 (12)	0 (0)	0 (0)
1 TAMs	1 (6)	0 (0)	0 (0)
2 TAMs	1 (6)	0 (0)	0 (0)
3+ TAMs	0 (0)	0 (0)	0 (0)
**41L**	0 (0)	0 (0)	0 (0)
**65R**	1 (6)	1 (8)	1 (10)
**67N**	1 (6)	0 (0)	0 (0)
**70R**	1 (6)	0 (0)	0 (0)
**184I/V**	7 (44)	8 (62)	4 (40)
**210W**	0 (0)	0 (0)	0 (0)
**215F/Y**	1 (6)	0 (0)	0 (0)
**NNRTI mutations**			
Any NNRTI mutation	8 (50)	9 (69)	6 (60)
90I	0 (0)	0 (0)	0 (0)
98G	0 (0)	0 (0)	0 (0)
**101E**	1 (6)	0 (0)	0 (0)
**103N**	3 (19)	3 (23)	3 (30)
108I	0 (0)	0 (0)	2 (20)
138A/G	0 (0)	0 (0)	0 (0)
179D	0 (0)	0 (0)	0 (0)
**181C/I**	0 (0)	4 (31)	2 (20)
**188L/H**	0 (0)	1 (8)	0 (0)
**190A/S**	3 (19)	2 (15)	1 (10)
**225H**	0 (0)	0 (0)	0 (0)
**230L**	0 (0)	0 (0)	0 (0)

Bold: major mutations. VLM: Virologically monitored; NRTI: nucleoside reverse transcriptase inhibitor; TAMs: thymidine analogue mutations; NNRTI: non-nucleoside reverse transcriptase inhibitor.

**Figure 2:**
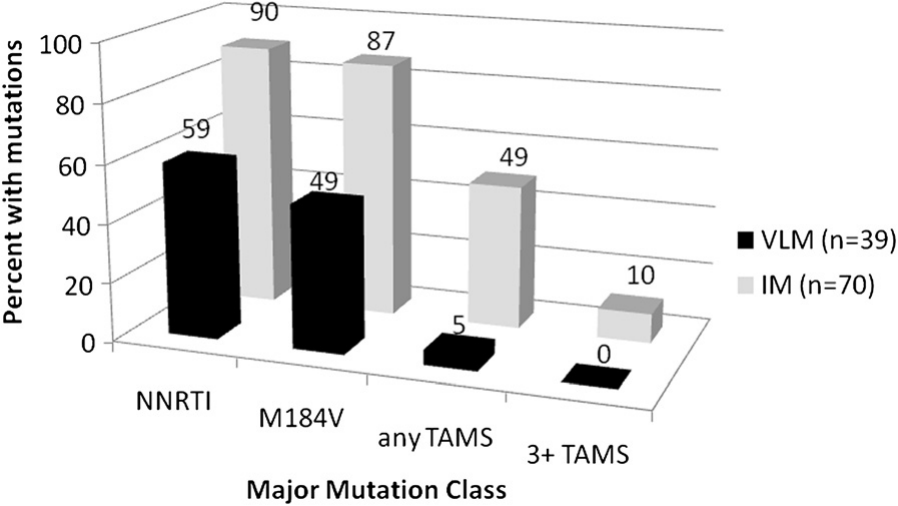
**Resistance by monitoring strategy.** VLM: Viral load monitoring; IM: Immunological monitoring; NNRTI: Non-nucleoside reverse transcriptase inhibitor; TAMS: Thymidine analogue mutations.

Comparing the 39 first genotypes from VLM clients to the 70 IM clients, 23/39 (59%) of VLM clients as opposed to 63/70 (90%) in the IM group had at least 1 NNRTI mutation (p < 0.0001) (Table 3). Nineteen out of thirty-nine (49%) of VLM clients had an M184V mutation compared to 61/70 (87%) of IM clients (P < 0.0001). Only 2/39 (5%) of the VLM clients developed TAMS whereas 34/70 (49%) of IM clients developed TAMS (p < 0.001) with 7/70 (10%) developing > = 3 TAMS (Figure 2).

**Table 3:** Resistance comparing IM versus VLM patients

	**IM**	**VLM**
	**(N = 70)**	**(N = 39)**
**NRTI mutations**		
Any TAMs	34 (49)	2 (5)
1 TAMs	13 (19)	1 (3)
2 TAMs	14 (20)	1 (3)
3+ TAMs	7 (10)	0 (0)
**41L**	8 (11)	0 (0)
**65R**	1 (1)	3 (8)
**67 N**	11 (16)	1 (3)
**70R**	13 (19)	1 (3)
**184I/V**	61 (87)	19 (49)
**210W**	1 (1)	0 (0)
**215F/Y**	25 (36)	1 (3)
**NNRTI mutations**		
Any NNRTI mutation	63 (90)	23 (59)
90I	5 (7)	0 (0)
98 G	5 (7)	0 (0)
**101E**	8 (11)	1 (3)
**103N**	23 (33)	10 (26)
108I	10 (14)	2 (5)
138A/G	3 (4)	0 (0)
179D	1 (1)	0 (0)
**181C/I**	22 (31)	6 (15)
**188L/H**	3 (4)	1 (3)
**190A/S**	13 (19)	6 (15)
**225H**	6 (9)	0 (0)
**230L**	1 (1)	0 (0)

Bold: major mutations.IM: Immunologically monitored; VLM: Virologically monitored; NRTI: nucleoside reverse transcriptase inhibitor; TAMS: thymidine analogue mutations; NNRTI: non-nucleoside reverse transcriptase inhibitor.

We also compared the 10 VLM clients with genotype results at 36 months to the 70 IM clients at 36 months, 6/10 (60%) of VLM clients as opposed to 63/70 (90%) in the IM group had at least 1 NNRTI mutation (p = 0.1034). Four out of ten (40%) of VLM clients had an M184V mutation compared to 61/70 (87%) of IM clients (P = 0.0185). None of the VLM clients developed TAMS whereas 34/70 (49%) of IM clients developed TAMS (p < 0.0001).

A total of 26 clients in the VLM cohort were switched from first to second-line ART in the first 36 months of follow-up, while all patients in the IM group reached 36 months on first-line ART without clinicians suspecting treatment failure. The median number of VL measurements before switch was 4 and median VL prior to switch was 21,773 (IQR 5,104-210,918).

## Discussion

Although the proportion of clients failing ART at 36 months was comparable between those monitored with VL and compared to those monitored with CD4+ cell counts only, the proportion of the clients with multiple resistance mutations especially TAMS was significantly higher in the IM clients. This is the first observational study directly comparing genotypic resistance profiles to commonly used antiretroviral drugs between individuals monitored with virologic compared to immunologic monitoring alone. Several studies have documented the lack of sensitivity of immunologic and clinical criteria to identify individuals failing ART raising concern regarding the potential for increased genotypic resistance to ART, which could result [3-6]. High rates of genotypic resistance to first-line ART have been observed in Malawi, Uganda and India where clinical and immunologic criteria have been used to identify treatment failure [8,11,18]. In addition, poor outcomes following first-line regimen failure have been documented in Malawi possibly due to advanced immunosuppression and ART resistance [19]. The results of our study add more evidence to the growing body of literature supporting some degree of viral load monitoring in RLS.

The participants enrolled in the IM cohort in this study were clients who had not been identified as having any evidence of treatment failure by clinicians. In contrast to most studies looking at resistance among clients in RLS, this was not a selected group of individuals failing by immunologic or clinical criteria and would have been left on first-line ART if VL testing was not done as part of this study. The significant degree of resistance observed in this group raises concern about the need for periodic VL monitoring in settings where none is currently available in routine care. A moderate proportion of clients had multiple TAMS possibly resulting in compromised efficacy of commonly used second-line ART regimens available in RLS.

Our study has limitations; it is not a randomized clinical trial and therefore unmeasured bias between the VLM and IM groups remains a possibility. Because the IM group was cross-sectional, we have no data on lost to follow-up or number of deaths before study enrollment; however, we would expect clients lost to care to do as poorly or worse with respect to rates of virologic failure. Clients enrolled in the VLM group followed certain inclusion criteria including living within 20 km of the clinic and willingness to attend to visit schedules which could also select for a more adherent population compared to the IM group. The use of stored specimens resulted in some amplification failures among the VLM group, which could possibly affect our overall results. Finally, we have no information on duration of virologic failure among the IM group to allow us to control for duration of failure which could influence the mutation patterns observed.

Despite the limitations mentioned pertaining to study design, baseline characteristics, treatment setting and study staff were all similar. Virologic and immunologic characteristics at 36 months of follow-up were also similar between the two groups. . We believe the reduced rate of resistance, particularly to NRTIs, observed among VLM clients is explained by the frequent VL monitoring and ability to detect and allow caregivers to discuss adherence and possibly switch to second-line regimens prior to the development of extensive drug resistance. Our results support ongoing efforts to improve adherence counseling strategies as treatment programs continue to scale up access. Our study was not designed to look at longer term mortality benefits between our VLM and IM clients, however, a recent multi-country study conducted in Malawi, Zambia and South Africa suggested that the lower rate of mortality observed among South African ART programs could be explained in part by the presence of VL monitoring and timely switch to second-line regimens [20]. As ART programs continue to scale up in RLS and funding constraints limit the choices of monitoring options, the debate over how best to monitor clients will continue. Some controlled clinical trials have failed to show a benefit of VL monitoring over immunologic or clinical monitoring [21,22]. Retention and adherence in these clinical trials is clearly higher than in the operational context of this study and may not be generalizable to operational contexts. Cost benefit studies have also suggested that allocating resources to earlier initiation of ART could provide more benefit than routine VL monitoring [23]. We feel it is equally important to evaluate monitoring strategies in a real world setting outside the support of a clinical trial where participants receive maximum adherence support and therefore risks of virologic failure are minimized. It is also important not to consider only an all or none approach to VL monitoring and consider new strategies using periodic VL monitoring at fixed time points during follow-up as a recent study in Thailand found cost-effective among pediatric clients [24].

## Conclusions

Access to viral load monitoring remains a priority for countries continuing to scale up ART in order to limit the emergence of drug resistance. Further studies are warranted to evaluate novel monitoring strategies that minimize resistance, maximize positive clinical outcomes, recognizing cost constraints faced by programs challenged by ever increasing numbers needing HIV treatment.

## Competing interests

The authors declared that they have no competing interests.

## Authors’ contributions

SJR, HK, TCQ and AK contributed to study design, participant recruitment and follow-up, and final analysis. KN contributed to study conduct and statistical analysis. YCM, MK and BC contributed to participant recruitment, study conduct and analysis. In performed laboratory assays and contributed to analysis. All authors contributed to manuscript writing. All authors read and approved the final manuscript.

## Pre-publication history

The pre-publication history for this paper can be accessed here:

http://www.biomedcentral.com/1471-2334/12/381/prepub
